# Reproductives and eggs trigger worker vibration in a subterranean termite

**DOI:** 10.1002/ece3.6325

**Published:** 2020-06-07

**Authors:** Fanny Ruhland, Marion Moulin, Marina Choppin, Joël Meunier, Christophe Lucas

**Affiliations:** ^1^ Institut de Recherche sur la Biologie de l’Insecte (UMR7261) CNRS – University of Tours Tours France

**Keywords:** body‐shaking, *Reticulitermes flavipes*, social behavior, termite, vibratory communication

## Abstract

In insect societies, the presence of reproductives or eggs has been shown to shape several biological traits in the colony members. Social interactions are one of these traits that involve modification of the communication system of the entire colony. Many studies described the role of chemical compounds and dominance behaviors in the presence of reproductive but vibratory behaviors received very few investigations. Yet, vibratory behaviors are ideal candidates, particularly for subterranean species like termites, as they could be quickly transmitted through the substrate and could be very diversified (origin, modulation). Here, we investigated whether the presence of reproductives/eggs affects the vibratory behavior (body‐shaking) of workers in the subterranean termite *Reticulitermes flavipes*. Our results reveal that the presence of reproductives or eggs triggers an increase of workers' body‐shaking, independent of their colony of origin after 24 hr. We hypothesize that vibratory communication could be used to transfer information about the presence of reproductives and eggs to the entire colony, suggesting that vibratory behaviors could serve as an important yet neglected mediator of social regulation.

## Graphical abstract

In the termite *Reticulitermes flavipes*, a modification of the social composition (presence/absence of reproductives and/or eggs) triggers a vibratory behavior in workers. This behavior is widespread among termites and was described so far as an alarm behavior. This brings new insight into the overlooked role of vibratory communication as a mediator of social regulation in insects.

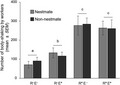

## 1 INTRODUCTION

In social insects, reproductives have a central role in colony functioning, since they can disperse, found new colonies, and ensure the production of new colony members. The other colony members are specialized in various tasks, such as defense, brood and reproductive care, foraging, and nest maintenance. Therefore, a modification in the social composition of the colony, such as the absence of reproductives, can strongly affect the social organization and therefore modify behaviors, gene expressions, and social regulation, sometimes resulting in the emergence of new reproductive members (Manfredini et al., 2014; Monnin & Ratnieks, 2001; Penick, Trobaugh, Brent, & Liebig, 2013).

Several studies highlighted modifications in workers' behavior in response to the presence or absence of reproductives. The queen can attract workers, enhance their foraging behavior, or even initiate antennation and grooming toward it (Herbers & Choiniere, 1996; Liebig, Eliyahu, & Brent, 2009). Interestingly, the effect of queen presence on worker behaviors may also be mediated by the presence of their eggs. In the termite *Reticulitermes speratus*, Matsuura et al. (2010) showed that eggs attract workers and that their presence inhibits worker differentiation into reproductives, an effect that is also present in ants (Endler et al., 2004). On the contrary, the absence of reproductives can imply more dominant or aggressive behavior displayed between workers (Korb, Weil, Hoffmann, Foster, & Rehli, 2009; Monnin & Ratnieks, 2001).

The display of vibratory behaviors is a common phenomenon among insects and especially social species. Cocroft and Rodríguez (2005) ascertained that almost eighty percent of the 195.000 insect species described use substrate‐born vibrations. In this way, some studies reported the display of vibratory behaviors of workers near reproductives or larvae in honey bees (Schneider & Lewis, 2004), *Polistes* (Suryanarayanan, Hermanson, & Jeanne, 2011)*,* or ants (Holldobler & Maschwitz, 1965). Despite the overall display of vibratory behaviors in social insects, this kind of observation is still scarce and we know no studies reporting the display of vibratory behaviors in the presence of eggs in eusocial species. The evidence exists, however, in the subsocial burrower bug *Adomerus rotundus* where female vibrate its abdomen in contact with egg mass (Mukai, Hironaka, Tojo, & Nomakuchi, 2012).

In termites, evidence of the display of vibratory behavior is inexistent in the presence/absence of eggs and rare in the presence/absence of reproductives despite the large utilization of substrate‐born vibrations in social interactions (Whitman & Forschler, 2007). Recent reports observed that a cuticular hydrocarbon presents on termite reproductive females induced more antennation and lateral oscillatory movements in workers (Funaro, Böröczky, Vargo, & Schal, 2018; Funaro, Schal, & Vargo, 2019). Numerous vibratory behaviors have been described in Isoptera and defined according to the way they are produced: vertical, complex, lateral, or longitudinal oscillatory movements (Howse, 1965; Whitman & Forschler, 2007). The longitudinal oscillatory movements (LOM), differently named among years and studies (body‐shaking, shaking, tremulation, longitudinal vibrations, jerking, jittery movements, jigging, trembling), are described in 5 of the 7 termite families (Hertel, Hanspach, & Plarre, 2011; Maistrello & Sbrenna, 1996; Ohmura, Takanashi, & Suzuki, 2009; Reinhard & Clément, 2002; Rosengaus, Jordan, Lefebvre, & Traniello, 1999; Šobotník, Hanus, & Roisin, 2008; Whitman & Forschler, 2007). It is defined as a rapid back and forth longitudinal movement of the whole body with no contact with the substrate and does not present any recipient (Whitman & Forschler, 2007). Whereas LOM, called body‐shaking in this study, have been mainly identified as a general alarm behavior (Hertel et al., 2011; Howse, 1965; Reinhard & Clément, 2002; Rosengaus et al., 1999), several studies suggest that body‐shaking can be involved in interindividual communication (reduction of cannibalistic grooming, dispersion) and be displayed spontaneously in association with defecation (Rosengaus et al., 1999; Whitman & Forschler, 2007).

In this study, we used the subterranean termite *Reticulitermes flavipes* to explore whether the presence/absence of reproductives and/or eggs entails changes in workers' body‐shaking. Because this vibratory behavior has been shown to be involved in the alarm signal, we also test whether the colony of origin of the workers could modulate the expression of body‐shaking. In France, *R. flavipes* is considered as an invasive species where colonial fusion occurs, resulting in acceptance of non‐nestmate members (Grace, 1996); therefore, we predict that the body‐shaking will not be affected by the colony of origin of the reproductives or eggs.

## 2 MATERIAL AND METHODS

### 2.1 Study species and laboratory conditions

Stock colonies of *R. flavipes* were collected from 2014 to 2017 in the Oléron island and near areas (Charente‐Maritime, France) with at least 300 m of distance between colonies (i.e., distances that typically ensure colony independency in this area) (Perdereau, Bagnères, Dupont, & Dedeine, 2010). In the laboratory, colonies were maintained under dark conditions at 26 ± 1°C with 95 ± 5% relative humidity within black plastic boxes. Each colony was kept in independent plastic boxes (Star‐pack) containing cellulosic ultrapure papers (47 mm diameter; Whatman, grade 42 Ashless) (Lucas et al., 2018) and supplied with wood sawdust.

### 2.2 Experimental setup and behavioral measurements

**FIGURE 1:**
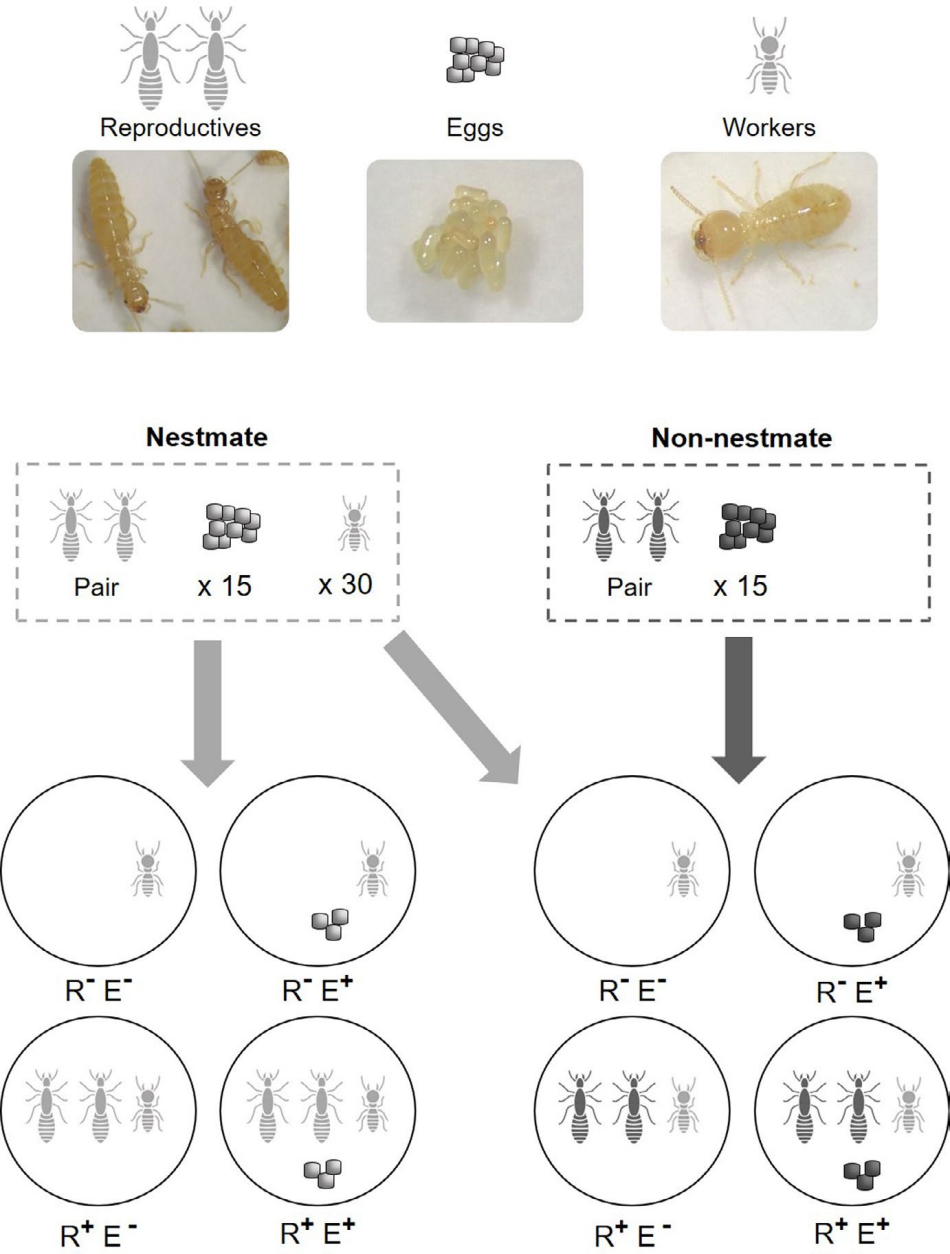
Experimental design representing different treatments (*n* = 15). Workers in the presence of reproductives and eggs (R^+^E^+^), in the presence of reproductives (R^+^E^‐^), in the presence of eggs (R^−^E^+^), and in the absence of reproductives and eggs (R^−^E^−^). Nestmate and non‐nestmate conditions are represented in different colors

Thirty workers isolated from 15 stock colonies were distributed among 4 types of experimental micronests either (a) together with both brachypterous reproductives (male and female) and eggs (R^+^E^+^; i.e., “R” for Reproductives and “E” for eggs; “+” for presence and “−” for absence), (b) together with both reproductives but no eggs (R^+^E^−^), (c) without any reproductives but with eggs (R^‐^E^+^), or (d) without any reproductives or eggs (R^−^E^−^) (for a total of 15 replicates per treatment; Figure 1). These four combinations were set up using eggs and brachypterous reproductives originating from either (a) a different colony than the workers, called “non‐nestmate treatment,” or (b) from the same colony as the workers, called “nestmate treatment” (Figure 1). The 15 stock colonies used in this experiment are mature and contained individuals of every caste. Micronests were made with plastic boxes (50 mm diameter; Star‐pack) with a cellulosic ultrapure paper (47 mm diameter; Whatman, grade 42 Ashless) humidified with 300 µl of microfiltered water. Prior to their introduction in the micronests, each reproductive (king and queen) was sampled from the stock colonies and then sexed on a CO2 pad. These individuals were then randomly paired (one king with one queen) and transferred to the corresponding micronests. Similarly, eggs were collected in their stock colonies and then counted prior to their transfer to the micronests. Note that in the R^+^E^+^ combination, eggs and reproductives always came from the same colonies. Finally, groups of 30 workers were sampled in the stock colonies, sorted on a CO2 pad, and then randomly distributed in the corresponding micronests.

Twenty‐four hours after their setup, each micronest was transferred to a video recording setup where individuals could settle down for 5 min prior to the recording of a video of 5 min (Panasonic HC‐VXF990) in a randomized order and under infrared light. Videos were then analyzed using the freeware BORIS v.6.0.5 (Friard & Gamba, 2016) to count the total number of body‐shaking expressed by the entire group of workers (together) during the 5 min of video. Data were recorded and analyzed blindly regarding the treatments (Gamboa, Reeve, & Holmes, 1991).

### 2.3 Statistical analyses

All statistical analyses were conducted using the software r (version 3.5.1; www.r‐project.org). The number of body‐shaking were tested using a general linear mixed model (LMM), in which the presence/absence of reproductives, the presence/absence of eggs, the colony of origin (“Nestmate” or “Non‐nestmate”), and all interactions among these three factors were used as explanatory factors. The stock colonies were also included as a random factor in the model to control for their multiple uses across the experimental micronests. To fit with homoscedasticity and normal distribution of model residuals, the numbers of “body‐shaking” were log‐transformed. The model was simplified following a step‐by‐step procedure removing the nonsignificant interactions, and then, post hoc Tukey HSD comparisons were conducted. Because the majority of reproductives did not perform body‐shaking (almost 92% of them) and were present in only two of the four treatments, the effects of “the presence of reproductives and/or eggs” and of “the colony of origin” on those measurements have been analyzed with the nonparametric Mann–Whitney. Body‐shaking of reproductives is expressed as mean ± SE.

## 3 RESULTS

**FIGURE 2:**
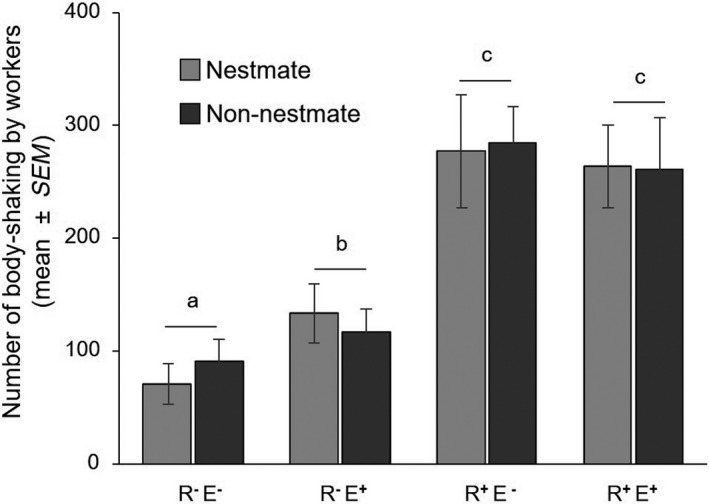
Number of body‐shaking displayed by 30 workers in the different treatments (mean ± *SEM*; *n* = 15). Workers in the presence of reproductives and eggs (R^+^E^+^), in the presence of reproductives (R^+^E^−^), in the presence of eggs (R^−^E^+^), and in the absence of reproductives and eggs (R^−^E^−^). Nestmate and non‐nestmate conditions are represented in different colors. Letters indicate significant differences between treatments (LMM, α = .05)

There is a significant interaction of the presence of reproductives and eggs on body‐shaking display (*F*_(4,115)_ = 6.161, *p* = .014; Figure 2). Post hoc tests show that workers displayed more body‐shaking in the presence of reproductives alone (*t*_(116)_ = 7.430, *p* < .001), eggs alone (*t*_(116)_ = 3.092, *p* = .013), and reproductives and eggs (*t*_(116)_ = 7.000, *p* < .001) than when they were absent. The presence of reproductives (*t*_(116)_ = 4.339, *p* < .001) or reproductives and eggs (*t*_(116)_ = 3.908, *p* < .001) induced significantly more body‐shaking display from workers than in the presence of eggs alone. The body‐shaking of workers was not significantly different in the presence of eggs and reproductives compared to the presence of reproductives alone (*t*_(116)_ = 0.430, *p* = .973).

Interestingly, we noticed that reproductives do also display body‐shaking but it did not vary with the presence of eggs (W = 13, *p* = .671; R^+^E^−^: 0.73 ± 0.42, R^+^E^+^: 0.27 ± 0.13) or with the colony of origin (R^+^E^‐^: W = 454, *p* = .936, “nestmate”: 0.80 ± 0.60, “non‐nestmate”: 0.67 ± 0.60; R^+^E^+^: W = 1684, *p* = .206, “nestmate”: 0.33 ± 0.16, “non‐nestmate”: 0.20 ± 0.20).

The body‐shaking is independent from the colony of origin of reproductives and eggs (*F*_(4,115)_ = 0.241, *p* = .624; Figure 2). There is no interaction between the colony of origin of reproductives/eggs and the presence of reproductives and/or eggs (all *p* > .260).

The mortality of workers after 24 hr did not vary with the presence/absence of reproductives and eggs or the colony of origin and or any interaction among these three factors (all *p* > .239). None of the reproductives died during this experiment.

## 4 DISCUSSION

This study demonstrates that the presence of reproductives triggers a strong expression of vibratory behavior by *R. flavipes* workers after 24 hr (around 3 times more than in the absence of reproductives). Moreover, eggs trigger a significant increase of body‐shaking in the absence of reproductives with no additive effect in the presence of reproductives. Those observed effects are independent of the colony of origin. Supplementary measurements indicate that reproductives display body‐shaking behaviors at a very low rate which is independent of the conditions (data not shown). Moreover, no mortality differences are observed between treatments either for workers or for reproductives (Figure S1).

The presence of reproductives and/or eggs induced a behavioral response of *R. flavipes* workers with an increased number of body‐shaking after 24 hr. The absence of reproductives has been studied in two termite species, *Zootermopsis nevadensi*s (Penick et al., 2013) and *Cryptotermes secundus* (Korb et al., 2009), where workers display more dominant behavior (head‐butting) in the absence of reproductives. The most aggressive workers (displaying more head‐butting) are the ones who will differentiate into reproductives, to replace the absent queen, highlighting its potential role into reproductive regulation (Korb et al., 2009). Different vibratory behaviors have been associated with the presence of reproductives in several social insect species like in honey bees (Schneider & Lewis, 2004) or other termite species (Funaro et al., 2018, 2019), which confirm our results here. Concerning the presence of eggs, in the sister species, *R. speratus*, an egg recognition pheromone (TERP) has been identified on the egg surface, attracting workers and initiating care behaviors (Matsuura et al., 2010; Matsuura, Tamura, Kobayashi, Yashiro, & Tatsumi, 2007). It could be interesting to test whether the body‐shaking, displayed in the presence of eggs, is dependent on the number of eggs and whether it could be initiated by egg chemical compounds. Interestingly, our results show that there was a nonadditive effect of the presence of eggs with the presence of reproductives on workers' vibratory behavior after 24h. This result is in accordance with the only study investigating the absence of eggs effect on the behavior of termite workers, showing that the inhibitory effect elicited by the queen on new queen differentiation is not reduced by egg removal (Matsuura et al., 2010). Our results show for the first time that modification of the social structure (the presence of reproductives and eggs on their own) triggers an increase of the shaking vibratory behavior of termite workers, which brings new insights into the potential role of this vibratory behavior in social organization. It will be interesting to investigate whether this could be generalized to the other types of reproductives (primary or apterous reproductives) with different social structures. Indeed, our current investigations were done on brachypterous reproductives isolated from mature colonies presenting all castes. A first answer can be found in incipient colonies, where it has been shown that primary reproductives modulate their body‐shaking according to the presence of eggs or descendants over a 6‐month period (Brossette, Meunier, Dupont, Bagnères, & Lucas, 2019). Again, it emphases the potential role of the body‐shaking in social organization and highlights the question about the role of vibratory behavior in social regulation. To understand this potential role in social regulation, we must keep in mind that the increase of body‐shaking in our experiments was present at 24h after the setup of the micronests. Therefore, the question raises about the dynamic of the behavioral answer overtime and its stability with variations in the number of reproductives.

According to the literature, several roles could be attributed to the display of body‐shaking in the presence of reproductives/eggs. First, it could represent a proxy for the fertility status of the colony and therefore could play a role in the regulation process to access reproduction. Indeed, in some social species, several vibratory behaviors have been observed to be linked with larval differentiation and reproductive regulation (Jeanne, 2009; Mignini & Lorenzi, 2015). In the termite *Cryptotermes domesticus,* the worker exposure to the full vibrational activities of a colony including chewing, walking, and oscillatory movements induces less reproductive differentiation (Evans et al., 2005). Alternately, body‐shakings produced by workers in the presence of reproductives/eggs could also represent a recruitment signal performed by workers to enroll nestmates in order to assist them and ensure appropriate care for reproductive and eggs. Indeed, workers conduct many essential tasks for the colony's development and especially they provide care to the reproductive (trophallaxy, grooming…) and eggs (grooming, egg pill formation …). In several insect societies, vibratory communication has been associated with recruitment and the global increase of the colony members’ activity (Pielström & Roces, 2012; Tautz, 1996). In termites, head‐drumming has been shown to attract nestmates to the source of disturbance (Reinhard & Clément, 2002; Stuart, 1963), and in *Cryptotermes secundus*, workers are attracted by vibratory signals resulting from chewing and walking (Evans, Inta, Lai, & Lenz, 2007). Overall, direct demonstrations of the potential function of the different vibratory sources on the social organization remain to be identified, including the body‐shaking.

Finally, this study shows that the colony of origin of reproductives and eggs did not modify the body‐shaking behavior of workers after 24 hr. In insect societies, the detection of intruders can result in the alarm and aggressive behaviors excluding non‐nestmate individuals and causing injuries (Haverty, Copren, Getty, & Lewis, 1999). Interestingly, vibratory signals and especially body‐shaking were originally described as an alarm behavior in termites (Hertel et al., 2011; Reinhard & Clément, 2002). In species with stricter colony boundaries, we could expect differences in body‐shaking according to the colony of origin, with more vibratory behaviors in the presence of non‐nestmates. But *R. flavipes* being an invasive species in France, it went through a population bottleneck potentially leading to unicoloniality resulting in low aggressivity. Our results are therefore in accordance with an open recognition system in *R. flavipes* leading to the performance of colonial fusion with low aggressive interactions (Clément, 1986; Grace, 1996; Perdereau et al., 2010). However, we can also make the hypothesis that workers do not show discrimination against non‐nestmate reproductives due to their fertile status, inducing acceptance despite the alien signal they could carry. Indeed, non‐nestmate reproductives and/or eggs could be considered as valuable enough to be accepted, inducing fitness benefits despite the potential costs associated with the acceptation of intruders (i.e., parasitism risks) (Matsuura & Nishida, 2001; Simkovic, Thompson, & McNeil, 2018).

Vibratory communication is widespread in insect societies, but its social role remains poorly explored. Our results bring new hypotheses on the function of a vibratory cue called body‐shaking. We show that it could be used as an efficient signal to communicate information about modifications of the colony social organization or to help nestmates to localize and focus on reproductives and eggs, two main valuable items of the colony. Body‐shaking has already been observed to have several functions, mainly as alarm signal (Hertel et al., 2011; Howse, 1965; Reinhard & Clément, 2002; Rosengaus et al., 1999), but this diversity of functions assigned to one behavioral item could be more complex than previously expected. We hypothesize that variability in its physical specifications could hide different functions with different behavioral effects. A brief description has already been made in abiotic conditions but not in different social contexts (Howse, 1965; Whitman & Forschler, 2007). In the future, more accurate measurements of this vibratory behavior need to be undertaken in different contexts in order to identify the modulation processes and their roles in social organization.

## CONFLICT OF INTEREST

The authors declare that they have no conflict of interests.

## AUTHOR CONTRIBUTION

**Fanny Ruhland:** Formal analysis (equal); Investigation (supporting); Methodology (equal); Project administration (supporting); Visualization (equal); Writing‐original draft (lead); Writing‐review & editing (equal). **Marion Moulin:** Investigation (equal); Methodology (supporting); Writing‐original draft (supporting). **Marina Choppin:** Investigation (equal); Methodology (supporting); Writing‐original draft (supporting). **Joël Meunier:** Formal analysis (equal); Writing‐original draft (supporting); Writing‐review & editing (supporting). **Christophe Lucas:** Conceptualization (lead); Funding acquisition (lead); Methodology (equal); Project administration (lead); Resources (lead); Supervision (lead); Visualization (equal); Writing‐original draft (equal); Writing‐review & editing (equal).

## Supporting information

Fig S1

## Data Availability

Data supporting the results are available in Dryad repository (https://doi.org/10.5061/dryad.7sqv9s4pv).

## References

[ece36325-bib-0001] Brossette, L. , Meunier, J. , Dupont, S. , Bagnères, A.‐G. , & Lucas, C. (2019). Unbalanced biparental care during colony foundation in two subterranean termites. Ecology and Evolution, 9(1), 192–200. 10.1002/ece3.4710 30680106 PMC6342128

[ece36325-bib-0002] Clément, J.‐L. (1986). Open and closed societies in *Reticulitermes termites* (Isoptera, Rhinotermitidae): Geographic and seasonal variations. Open and Closed Societies in Reticulitermes Termites (Isoptera, Rhinotermitidae): Geographic and Seasonal Variations, 11(3), 311–323.

[ece36325-bib-0003] Cocroft, R. B. , & Rodríguez, R. L. (2005). The Behavioral Ecology of Insect Vibrational Communication. BioScience, 55(4), 323–334. 10.1641/0006-3568(2005)055[0323:TBEOIV]2.0.CO;2

[ece36325-bib-0004] Endler, A. , Liebig, J. , Schmitt, T. , Parker, J. E. , Jones, G. R. , Schreier, P. , & Hölldobler, B. (2004). Surface hydrocarbons of queen eggs regulate worker reproduction in a social insect. Proceedings of the National Academy of Sciences of the United States of America, 101(9), 2945–2950. 10.1073/pnas.0308447101 14993614 PMC365725

[ece36325-bib-0005] Evans, T. A. , Inta, R. , Lai, J. C. S. , & Lenz, M. (2007). Foraging vibration signals attract foragers and identify food size in the drywood termite, *Cryptotermes secundus* . Insectes Sociaux, 54(4), 374–382. 10.1007/s00040-007-0958-1

[ece36325-bib-0006] Evans, T. A. , Lai, J. C. S. , Toledano, E. , McDowall, L. , Rakotonarivo, S. , & Lenz, M. (2005). Termites assess wood size by using vibration signals. Proceedings of the National Academy of Sciences of the United States of America, 102(10), 3732–3737. 10.1073/pnas.0408649102 15734796 PMC553312

[ece36325-bib-0007] Friard, O. , & Gamba, M. (2016). BORIS: A free, versatile open‐source event‐logging software for video/audio coding and live observations. Methods in Ecology and Evolution, 7(11), 1325–1330. 10.1111/2041-210X.12584

[ece36325-bib-0008] Funaro, C. F. , Böröczky, K. , Vargo, E. L. , & Schal, C. (2018). Identification of a queen and king recognition pheromone in the subterranean termite *Reticulitermes flavipes* . Proceedings of the National Academy of Sciences of the United States of America, 115(15), 3888–3893. 10.1073/pnas.1721419115 29555778 PMC5899469

[ece36325-bib-0009] Funaro, C. F. , Schal, C. , & Vargo, E. L. (2019). Queen and king recognition in the subterranean termite, *Reticulitermes flavipes*: Evidence for royal recognition pheromones. PLoS ONE, 14(5), e0209810 10.1371/journal.pone.0209810 31145770 PMC6542537

[ece36325-bib-0010] Gamboa, G. J. , Reeve, H. K. , & Holmes, W. G. (1991). Conceptual issues and methodology in kin‐recognition research: A critical discussion. Ethology, 88(2), 109–127. 10.1111/j.1439-0310.1991.tb00267.x

[ece36325-bib-0011] Grace, J. K. (1996). Absence of overt agonistic behavior in a northern population of *Reticulitermes flavipes* (Isoptera: Rhinotermitidae). Sociobiology, 28(1), 103–110.

[ece36325-bib-0012] Haverty, M. I. , Copren, K. A. , Getty, G. M. , & Lewis, V. R. (1999). Agonistic behavior and cuticular hydrocarbon phenotypes of colonies of *Reticulitermes* (Isoptera: Rhinotermitidae) from Northern California. Annals of the Entomological Society of America, 92(2), 269–277. 10.1093/aesa/92.2.269

[ece36325-bib-0013] Herbers, J. M. , & Choiniere, E. (1996). Foraging behaviour and colony structure in ants. Animal Behaviour, 51(1), 141–153. 10.1006/anbe.1996.0012

[ece36325-bib-0014] Hertel, H. , Hanspach, A. , & Plarre, R. (2011). Differences in alarm responses in drywood and subterranean termites (Isoptera: Kalotermitidae and Rhinotermitidae) to physical stimuli. Journal of Insect Behavior, 24(2), 106–115. 10.1007/s10905-010-9240-x

[ece36325-bib-0015] Hölldobler, B. , & Maschwitz, U. (1965). Der Hochzeitsschwarm der Rossameise Camponotus herculeanus L. (Hym. formicidae). Zeitschrift für vergleichende Physiologie, 50(5), 551–568. 10.1007/BF00355658

[ece36325-bib-0016] Howse, P. E. (1965). On the significance of certain oscillatory movements of termites. Insectes Sociaux, 12(4), 335–345. 10.1007/BF02222723

[ece36325-bib-0017] Jeanne., (2009). Organization of Insect Societies: From Genome to Sociocomplexity. Cambridge, MA: Harvard University Press.

[ece36325-bib-0018] Korb, J. , Weil, T. , Hoffmann, K. , Foster, K. R. , & Rehli, M. (2009). A gene necessary for reproductive suppression in termites. Science, 324(5928), 758–758. 10.1126/science.1170660 19423819

[ece36325-bib-0019] Liebig, J. , Eliyahu, D. , & Brent, C. S. (2009). Cuticular hydrocarbon profiles indicate reproductive status in the termite *Zootermopsis nevadensis* . Behavioral Ecology and Sociobiology, 63(12), 1799–1807. 10.1007/s00265-009-0807-5

[ece36325-bib-0020] Lucas, C. , Brossette, L. , Lefloch, L. , Dupont, S. , Christidès, J.‐P. , & Bagnères, A.‐G. (2018). When predator odour makes groups stronger: Effects on behavioural and chemical adaptations in two termite species. Ecological Entomology, 43(4), 513–524. 10.1111/een.12529

[ece36325-bib-0021] Maistrello, L. , & Sbrenna, G. (1996). Frequency of some behavioural patterns in colonies of *Kalotermes flavicollis* (Isoptera Kalotermitidae): The importance of social interactions and vibratory movements as mechanisms for social integration. Ethology Ecology & Evolution, 8(4), 365–375. 10.1080/08927014.1996.9522909

[ece36325-bib-0022] Manfredini, F. , Lucas, C. , Nicolas, M. , Keller, L. , Shoemaker, D. , & Grozinger, C. M. (2014). Molecular and social regulation of worker division of labour in fire ants. Molecular Ecology, 23(3), 660–672. 10.1111/mec.12626 24329612

[ece36325-bib-0023] Matsuura, K. , Himuro, C. , Yokoi, T. , Yamamoto, Y. , Vargo, E. L. , & Keller, L. (2010). Identification of a pheromone regulating caste differentiation in termites. Proceedings of the National Academy of Sciences of the United States of America, 107(29), 12963–12968. 10.1073/pnas.1004675107 20615972 PMC2919916

[ece36325-bib-0024] Matsuura, K. , & Nishida, T. (2001). Colony fusion in a termite: What makes the society “open”? Insectes Sociaux, 48(4), 378–383. 10.1007/PL00001795

[ece36325-bib-0025] Matsuura, K. , Tamura, T. , Kobayashi, N. , Yashiro, T. , & Tatsumi, S. (2007). The antibacterial protein lysozyme identified as the termite egg recognition pheromone. PLoS ONE, 2(8), 1–9. 10.1371/journal.pone.0000813 PMC1950569 17726543

[ece36325-bib-0026] Mignini, M. , & Lorenzi, M. C. (2015). Vibratory signals predict rank and offspring caste ratio in a social insect. Behavioral Ecology and Sociobiology, 69(10), 1739–1748. 10.1007/s00265-015-1986-x

[ece36325-bib-0027] Monnin, T. , & Ratnieks, F. L. (2001). Policing in queenless ponerine ants. Behavioral Ecology and Sociobiology, 50(2), 97–108. 10.1007/s002650100351

[ece36325-bib-0028] Mukai, H. , Hironaka, M. , Tojo, S. , & Nomakuchi, S. (2012). Maternal vibration induces synchronous hatching in a subsocial burrower bug. Animal Behaviour, 84(6), 1443–1448. 10.1016/j.anbehav.2012.09.012

[ece36325-bib-0029] Ohmura, W. , Takanashi, T. , & Suzuki, Y. (2009). Behavioral analysis of tremulation and tapping of termites (Isoptera). Sociobiology, 54(1), 269–274.

[ece36325-bib-0030] Penick, C. A. , Trobaugh, B. , Brent, C. S. , & Liebig, J. (2013). Head‐butting as an early indicator of reproductive disinhibition in the termite *Zootermopsis nevadensis* . Journal of Insect Behavior, 26(1), 23–34. 10.1007/s10905-012-9332-x

[ece36325-bib-0031] Perdereau, E. , Bagnères, A.‐G. , Dupont, S. , & Dedeine, F. (2010). High occurrence of colony fusion in a European population of the American termite *Reticulitermes flavipes* . Insectes Sociaux, 57(4), 393–402. 10.1007/s00040-010-0096-z

[ece36325-bib-0032] Pielström, S. , & Roces, F. (2012). Vibrational communication in the spatial organization of collective digging in the leaf‐cutting ant *Atta vollenweideri* . Animal Behaviour, 84(4), 743–752. 10.1016/j.anbehav.2012.07.008

[ece36325-bib-0033] Reinhard, J. , & Clément, J.‐L. (2002). Alarm reaction of european reticulitermes termites to soldier head capsule volatiles (Isoptera, Rhinotermitidae). Journal of Insect Behavior, 15(1), 95–107. 10.1023/A:1014436313710

[ece36325-bib-0034] Rosengaus, R. B. , Jordan, C. , Lefebvre, M. L. , & Traniello, J. F. A. (1999). Pathogen alarm behavior in a termite: A new form of communication in social insects. Naturwissenschaften, 86(11), 544–548. 10.1007/s001140050672 10551951

[ece36325-bib-0035] Schneider, S. S. , & Lewis, L. A. (2004). The vibration signal, modulatory communication and the organization of labor in honey bees, *Apis mellifera* . Apidologie, 35(2), 117–131. 10.1051/apido:2004006

[ece36325-bib-0036] Simkovic, V. , Thompson, G. J. , & McNeil, J. N. (2018). Testing for aggression and nestmate recognition in the Eastern subterranean termite (*Reticulitermes flavipes*). Insectes Sociaux, 65(2), 281–288. 10.1007/s00040-018-0608-9

[ece36325-bib-0037] Šobotník, J. , Hanus, R. , & Roisin, Y. (2008). Agonistic behavior of the termite prorhinotermes canalifrons (Isoptera: Rhinotermitidae). Journal of Insect Behavior, 21(6), 521–534. 10.1007/s10905-008-9147-y

[ece36325-bib-0038] Stuart, A. M. (1963). Studies on the communication of alarm in the termite *Zootermopsis nevadensis* (Hagen), Isoptera. Physiological Zoology, 36(1), 85–96. 10.1086/physzool.36.1.30152740

[ece36325-bib-0039] Suryanarayanan, S. , Hermanson, J. C. , & Jeanne, R. L. (2011). A mechanical signal biases caste development in a social wasp. Current Biology, 21(3), 231–235. 10.1016/j.cub.2011.01.003 21256023

[ece36325-bib-0040] Tautz, J. (1996). Honeybee waggle dance: Recruitment success depends on the dance floor. Journal of Experimental Biology, 199(6), 1375–1381. 9319269 10.1242/jeb.199.6.1375

[ece36325-bib-0041] Whitman, J. G. , & Forschler, B. T. (2007). Observational notes on short‐lived and infrequent behaviors displayed by *Reticulitermes flavipes* (Isoptera: Rhinotermitidae). Annals of the Entomological Society of America, 100(5), 763–771. 10.1603/0013-8746(2007)100[763:ONOSAI]2.0.CO;2

