# Supplementary figures and images for: Reproductives and eggs trigger worker vibration in a subterranean termite
Source: Ecol Evol. 2020 Jun 7;10(12):5892–8. doi: 10.1002/ece3.6325 (PMC7319145; doi:10.1002/ece3.6325)

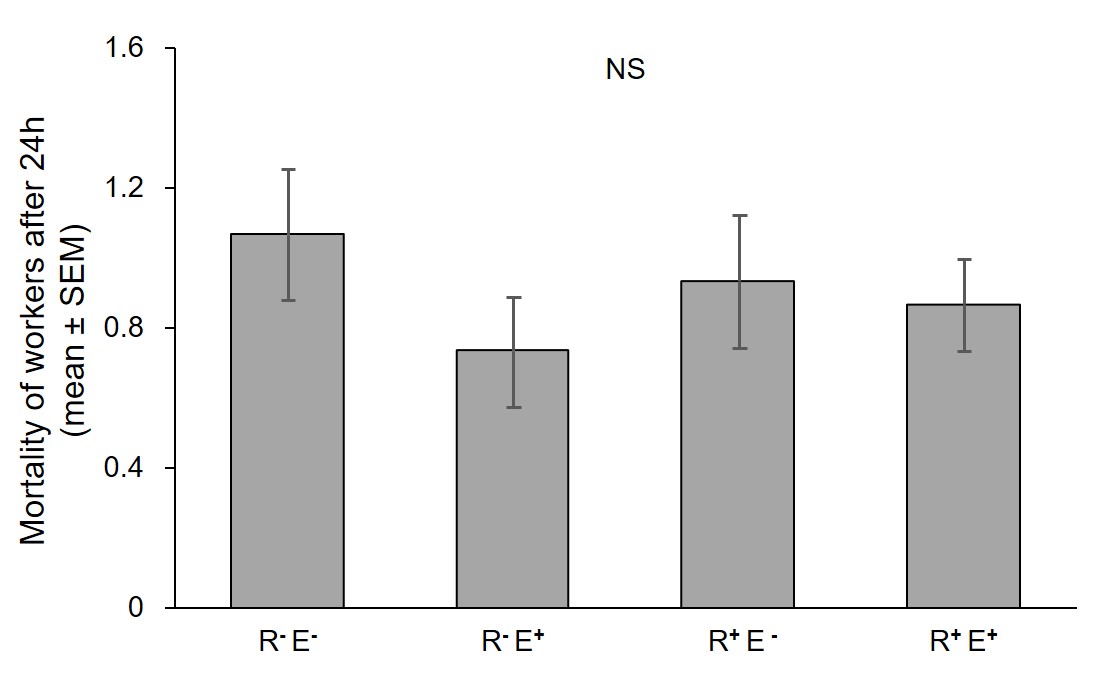

Supplement: Supplementary file 1 — Fig S1 [file ECE3-10-5892-s001.jpg]
